# Identification of endoxylanase XynE from *Clostridium thermocellum* as the first xylanase of glycoside hydrolase family GH141

**DOI:** 10.1038/s41598-017-11598-y

**Published:** 2017-09-11

**Authors:** Simon Heinze, Matthias Mechelke, Petra Kornberger, Wolfgang Liebl, Wolfgang H. Schwarz, Vladimir V. Zverlov

**Affiliations:** 1 Department of Microbiology, Technical University of Munich, Emil-Ramann-Str. 4, D-85354 Freising-Weihenstephan, Germany; 2 Institute of Molecular Genetics, Russian Academy of Science, Kurchatov Sq. 2, 123182 Moscow, Russia

## Abstract

Enzymes that cleave polysaccharides in lignocellulose, i. e., cellulases, xylanases, and accessory enzymes, play crucial roles in the natural decomposition of plant-derived biomass and its efficient and sustainable processing into biofuels or other bulk chemicals. The analysis of open reading frame cthe_2195 from the thermophilic, cellulolytic anaerobe *Clostridium thermocellum* (also known as *‘Ruminiclostridium thermocellum*’) suggested that it encoded a cellulosomal protein comprising a dockerin-I module, a carbohydrate-binding module, and a module of previously unknown function. The biochemical characterisation upon recombinant expression in *Escherichia coli* revealed that the protein is a thermostable endoxylanase, named Xyn141E with an optimal pH of 6.0–6.5 and a temperature optimum of 67–75 °C. The substrate spectrum of Xyn141E resembles that of GH10 xylanases, because of its side activities on carboxymethyl cellulose, barley β-glucan, and mannan. Conversely, the product spectrum of Xyn141E acting on arabinoxylan is similar to those of GH11, as established by HPAEC-PAD analysis. Xyn141E is weakly related (20.7% amino acid sequence identity) to the founding member of the recently established GH family 141 and is the first xylanase in this new family of biomass-degrading enzymes.

## Introduction

Research on carbohydrate-degrading enzymes has advanced markedly since their discovery in 1833^1, 2^, with the first descriptions of cellulases in 1912^3^ and of xylanases in 1955^4, 5^, as well as the publication of a comprehensive database of carbohydrate-active enzymes (CAZy) in 1998^6^. To date, more than 400,000 DNA sequences associated with enzymes targeting carbohydrates have been identified. These have been grouped into five classes: glycoside hydrolases (GH), glycosyl transferases (GT), polysaccharide lyases (PL), carbohydrate esterases (CE), auxiliary activities (AA), and associated carbohydrate-binding modules (CBM). The major class of carbohydrate-degrading enzymes, the GH, includes more than 330,000 enzymes, of which approximately 2% have been characterised. Based on structural similarities, GH enzymes have been grouped into 145 different families^6^. Although a vast number of proteins have been characterised and functionally annotated, many putative proteins have not yet been connected to a biological function. The interest in discovering new glycoside hydrolases continues to be high: for example, research on glycoside hydrolases gives insight into fields such as carbohydrate utilisation by human gut microbiota^7^ and can provide new functionalities for the efficient breakdown of plant biomass for industrial biotechnology. Further investigation of uncharacterised proteins will contribute to a better understanding and utilisation of carbohydrate-degrading microorganisms, as well as to the annotation of gene function in biological databases.

The complex structure of xylan, the second-most abundant polysaccharide^8^, has inspired the evolution of a sophisticated set of enzymes for its degradation. This toolbox includes endo-1,4-β-xylanases (EC 3.2.1.8) for the hydrolysis of the β-1,4-linked xylan backbone; α-l-arabinofuranosidases (EC 3.2.1.55) and α-d-glucuronidases (EC 3.2.1.139) for the liberation of α-arabinofuranosyl and 4-*O*-methyl-α-glucuronic acid moieties, respectively; specific esterases for the removal of acetate and ferulic acid esters (EC 3.1.1.72 and EC 3.1.1.73); and β-xylosidases (EC 3.2.1.37) for the degradation of xylo-oligosaccharides into xylose^9, 10^.

The key players in xylan depolymerisation are endoxylanases. The two GH families containing the majority of xylanases are GH10 and GH11, but enzymes that display xylanase activity are also found in other GH families (GH5, GH7, GH8, and GH43), with other activities or bifunctionalities with some activity against xylan also found in families GH16, GH51, GH52, and GH62^5^. The major xylanase families GH10 and GH11 differ from one another substantially^5, 10–12^. GH10 enzymes tend to have a higher molecular mass and lower pI than GH11 enzymes. GH10 enzymes are structurally defined by a (β/α)_8_ fold, whereas GH11 enzymes consist of three β-pleated sheets and one α-helix, forming a β-jelly roll^8, 13, 14^. GH10 xylanases exhibit high catalytic versatility as they can cleave the xylan backbone at the non-reducing side of xylose residues to which the arabinofuranosyl or 4-*O*-methyl-d-glucuronic acid side groups are attached. In contrast, GH11 xylanases can only cleave unsubstituted regions of the xylan backbone and are hindered by both arabinofuranosyl as well as 4-*O*-methyl-d-glucuronic acid side groups proximal to the site of cleavage. Overall, xylo-oligosaccharides produced by GH10 xylanases tend to be smaller and are more often arabinosylated than those produced by GH11 xylanases^5, 8, 10, 15^.

One of the first thermophilic bacteria from which xylanases were isolated is *Clostridium thermocellum* ^16–21^ (*‘Ruminiclostridium thermocellum’*, see ref. 22). This organism efficiently degrades plant biomass using the cellulosome, an extracellular protein complex that consists of enzymatic components attached to a noncatalytic scaffoldin via a cohesin-dockerin type I interaction. Hence, the individual components are placed in spatial proximity to one another, which improves the cooperation of enzymes with different mechanisms of action^23^. Catalytic efficiency can be further increased by association of the catalytic modules with a CBM, which improves substrate binding. Although *C. thermocellum* is limited to using cellodextrins as its carbon source, only 27 of the 71 cellulosomal components are categorised as β-glucanases^24, 25^. Among the other enzymes present in the cellulosome, there are a great number of hemicellulases, such as xylanases Xyn11A, Xyn10C, Xyn10D, Xyn10Y, and Xyn10Z. Other cellulosomal proteins with annotated non-catalytic modules have not been assigned a catalytic function [“components with unknown function” in ref. 25].

Here we report the characterisation of a cellulosomal protein from *C. thermocellum* with a previously unknown function. The gene product of open reading frame (ORF) cthe_2195, encoding a previously unknown module fused to CBM6 and dockerin I (DocI), was recombinantly produced in *E. coli* and characterised as an endoxylanase. The protein was named Xyn141E and is the first xylanase of family GH141, a new GH family published shortly before submission if this manuscript^7^.

## Results

### Sequence analysis

**Figure 1:**
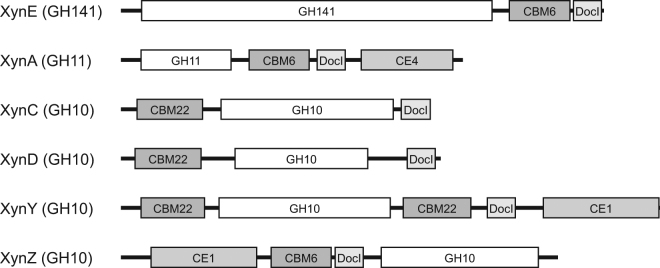
Overview of the module architectures of Xyn141E and other *C. thermocellum* xylanases of the GH10 and GH11 families. CBM: carbohydrate-binding module, DocI: dockerin I, CE: carbohydrate esterase, GH: glycoside hydrolase. The actual extent of the putative catalytic module (GH141) is currently not known precisely.

*C. thermocellum* ATCC 27405 ORF cthe_2195 (gene bank accession number ABN53397.1) encodes a polypeptide of 965 amino acids named Xyn141E. The first blastp search of the amino acid sequence of Xyn141E revealed three conserved modules. Both CBM6 (amino acids 772–894) and the type-I Doc-repeat domain (amino acids 903–959) are located at the C-terminal end of the protein. A putative signal peptide (amino acids 1–39) was predicted by SignalP 4.1^26^. Within the unknown region of the polypeptide, a right-handed beta-helix region (pfam13229) was predicted from amino acid residues 384 to 530, which according to Pfam annotation information “*shares some similarity with pectate lyases*.” Fig. 1 depicts an overview of the modules of Xyn141E in comparison with other *C. thermocellum* xylanases from the GH10 and GH11 families. The main ligand specificity of CBM6 is directed towards xylan, but other substrate specificities also can occur^27–29^. Since catalytic activity has not been observed for either DocI or CBM alone, the N-terminal region (amino acids 1–750) was used in a blastp search for related proteins (accessed January 16, 2017). While this search did not reveal any homology to previously characterised proteins, it did identify 204 homologous proteins with high significance (e Values < 1e-50) and sequence identities between 30% and 94%. The identified proteins originated from approximately 162 different bacteria across a broad range of genera and species. The most abundant phyla were *Actinobacteria* (n = 93) and *Firmicutes* (n = 44). Xyn141E is a member of family GH141, which was recently introduced with the description of BT1002 from *Bacteroides thetaiotaomicron* VPI-5482^6, 7^. BT1002 and Xyn141E share a sequence similarity of only 33.4% (identity 20.7%, pairwise global alignment using EMBOSS Needle with default settings, http://www.ebi.ac.uk/Tools/psa/emboss_needle/ ^30^). The catalytic amino acids of BT1002 (Asp523 and Asp564^7^) are conserved in Xyn141E and correspond to Asp540 and Asp577, respectively.

### Production and purification of Xyn141E

The pET24c(+)-cthe2195syn plasmid was used for the expression of *xynE* in *E. coli* BL21 Star™, followed by purification of the His-tagged protein from cell lysates using immobilised metal ion affinity chromatography (IMAC). Due to its origin from a thermophilic microorganism, inherent thermostability of Xyn141E was expected. This feature allowed for the use of heat precipitation as an additional purification step. SDS-PAGE analysis revealed a band corresponding to the expected molecular mass of 102.2 kDa, which appeared free of impurities.

### Substrate specificity

Xyn141E exhibited its primary activity against four different types of xylan (soluble wheat arabinoxylan, birch wood xylan, oat spelt xylan, and 4-*O*-methyl-glucuronoxylan) with relative activities between 49.8% and 100%. It was most active against soluble wheat arabinoxylan, with a specific activity of 92 ± 5 mU/mg (=100%). Additionally, it was also active against carboxymethyl cellulose (CMC), barley β-glucan (BBG), and ivory nut mannan (relative activities between 9.1% and 27.7%). The activities against these substrates are listed in Table 1. No activity against any of the other tested substrates, such as arabinan, different types of galactans, β-1,3-glucans, or pectins (Supplementary Table S1), was detected within the limits of accuracy of the experiment. The presence of side activities towards non-xylan substrates resembles the catalytic versatility of GH10 enzymes, suggesting a GH10-like substrate spectrum. In contrast to GH11 xylanases, GH10 xylanases have been reported to be active also against β-glucan, whereas GH11 xylanases are specific for xylan^5, 8, 14, 18^. The activity of Xyn141E against various *p*NP-glycosides (Supplementary Table S2) was also tested, but no significant activity was detected.

**Table 1:** Specific and relative activities of Xyn141E towards various substrates. Substrates were incubated with 66.7 µg/mL purified enzyme in standard reaction buffer (pH 6.5) at 65 °C for 60 min. Activities ± standard deviations were determined by measuring the release of reducing sugars. Each measurement was performed in triplicate.

Substrate	Specific Activity [mU/mg]	Relative Activity [%]
Wheat arabinoxylan (soluble)	92 ± 5	100.0 ± 5.0
Oat spelt xylan	83 ± 5	91.5 ± 5.2
4-*O*-Methyl-glucuronoxylan	56 ± 18	61.5 ± 19.5
Birchwood xylan	46 ± 2	49.8 ± 2.0
Ivory nut mannan	8 ± 6	9.1 ± 6.8
CMC	25 ± 4	27.7 ± 5.2
Barley β-glucan	11 ± 6	12.2 ± 7.1

### Influence of pH, temperature, and additives on activity

The influence of pH on the enzymatic activity of Xyn141E was tested for pH 5.0–7.5 at 65 °C, using wheat arabinoxylan as a substrate. Xyn141E was most active at a pH between 6.0 and 6.5, and retained at least 78.6% of its activity at pH 5.0–7.0. At pH 7.5, the relative activity was 48.3%.

Given the thermophilic nature of *C. thermocellum*, the effect of temperature on Xyn141E activity was determined between 50 °C and 90 °C. The highest activity under our assay conditions at pH 6.5 was detected between 67 °C and 75 °C (97.7–100%), indicating a plateau of maximum activity at a range of high temperatures. At 50 °C, the residual activity was 46.4%, which is similar to the relative activity of 38.4% observed at 82 °C. At 90 °C, the enzyme was inactive.

Table 2 displays an overview of the impact of certain salts and organic compounds on Xyn141E activity. The presence of either 10 mM CuSO_4_ or 10 mM SDS inhibited Xyn141E completely.

**Table 2:** Influence of salts and organic compounds on Xyn141E. Wheat arabinoxylan [0.5% (w/v)] was incubated with 66.7 µg/mL purified Xyn141E in 100 mM MOPS, pH 6.5, at 65 °C for 60 min in the presence of the indicated compounds. Relative activities ± standard deviations were determined by measuring the release of reducing sugars. n. d.: no activity detected. All assays were performed in triplicate.

Additive	Relative Activity [%]
CaCl_2_, 10 mM	101.5 ± 2.6
MgCl_2_, 10 mM	95.4 ± 13.8
CuSO_4_, 1 mM	11.1 ± 1.2
CuSO_4_, 10 mM	n. d.
EDTA, 1 mM	64.8 ± 6.6
EDTA, 10 mM	66.0 ± 0.9
Ethanol, 1%	59.6 ± 4.1
Ethanol, 5%	50.7 ± 8.6
SDS, 1 mM	79.5 ± 6.9
SDS, 10 mM	n. d.
No additives	100.0 ± 16.6

### Analysis of polysaccharide degradation products

**Figure 2:**
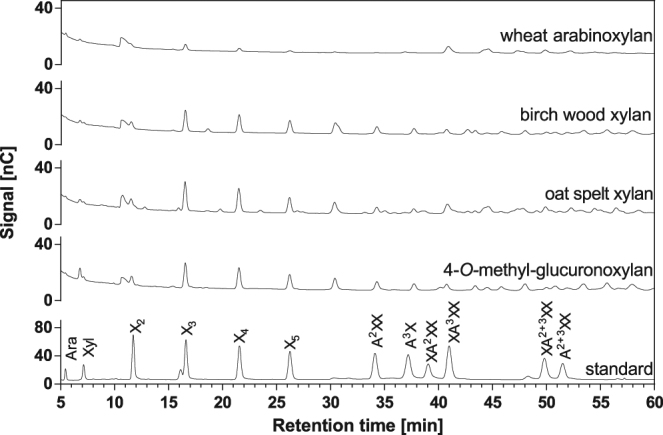
Product patterns of four types of xylan hydrolysed by Xyn141E and analysed by HPAEC-PAD. The indicated substrates [0.5% (w/v)] were incubated with 4 µg/mL Xyn141E for 1,200 min in standard reaction buffer (pH 6.5) at 60 °C. The standard represents a mixture of commercially available arabinoxylo- and xylo-oligosaccharides. X_2_, X_3_, X_4_, X_5_: xylobiose, -triose, -tetraose, and -pentaose, respectively. Arabinoxylo-oligosaccharides were named according to McCleary *et al*.^31^.

**Figure 3:**
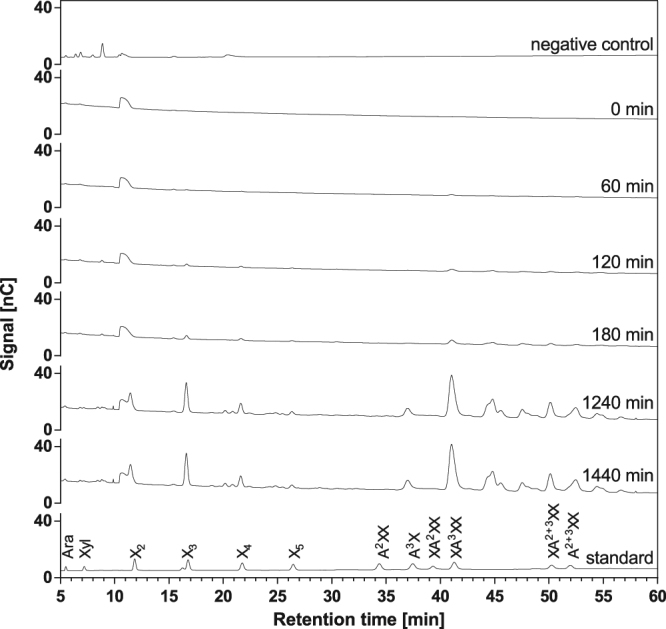
Time course of wheat arabinoxylan hydrolysis by Xyn141E. The substrate [0.5% (w/v)] was incubated with 200 µg/mL Xyn141E in standard reaction buffer (pH 6.5) at 65 °C for up to 1,440 min. At the indicated time points, samples were analysed by HPAEC-PAD. Negative control: no addition of Xyn141E.

The hydrolytic activity of Xyn141E was further verified by analysing the products released after overnight reactions with the four main substrates (Fig. 2). A comparison of peaks in the product chromatograms with standard substances allowed the identification of several oligosaccharides (Supplementary Table S3). In all cases, xylo-oligosaccharides (X_2_–X_5_) could be identified (retention times of 11.5 min–26.3 min). An additional peak observed at a retention time of 30.4 min may correspond to xylohexaose (X_6_). Starting at a retention time of 34.3 min, arabinoxylo-oligosaccharides were detected. While degradation products resembling most standard arabinoxylo-oligosaccharides were identified, XA^2^XX [nomenclature according to ref. 31] was missing. Additionally, there were a large number of small peaks for which reference substances were unavailable. To determine whether Xyn141E acts in an endo- or exo-mode, the emergence of individual products was followed over time using wheat arabinoxylan as a substrate (Fig. 3). The fact that all peaks start to appear at the same time (120 min) and continue to increase uniformly indicates that Xyn141E is clearly an endoxylanase.

### Comparison of the products of Xyn141E with those of other xylanases from *C. thermocellum*

**Figure 4:**
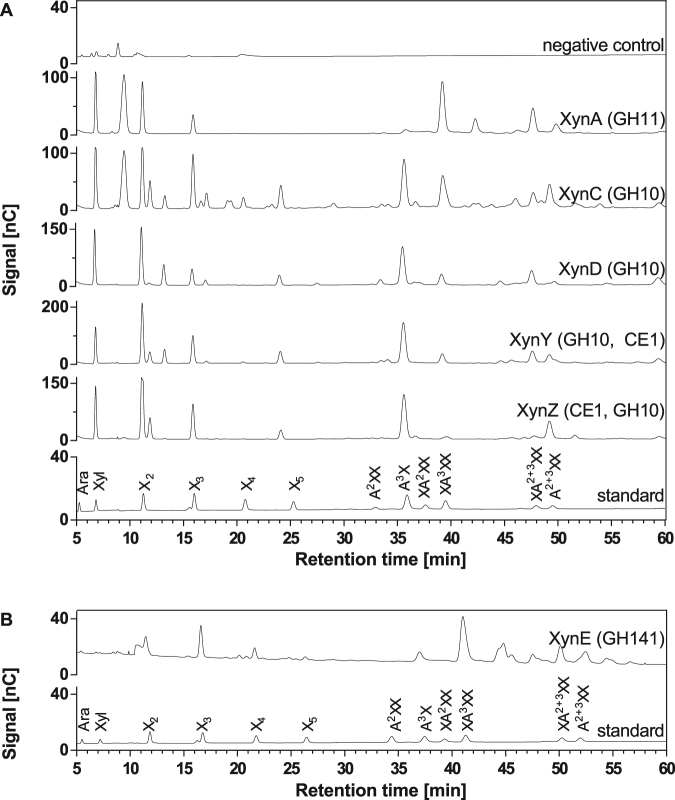
Comparison of product patterns of Xyn141E (**B**) and other xylanases from *C. thermocellum* (**A**). Purified enzymes were incubated overnight with 0.5% (w/v) wheat arabinoxylan in standard reaction buffer at (**A**) 60 °C (Xyn11A, Xyn10C, Xyn10D, Xyn10Y, and Xyn10Z) or (**B**) 65 °C (Xyn141E). Products were analysed by HPAEC-PAD. Negative control: 0.5% (w/v) wheat arabinoxylan incubated overnight in standard reaction buffer without the addition of enzyme.

*C. thermocellum* has five xylanase genes in xylanase families GH10 and GH11: Xyn11A (GH11, CE4), Xyn10C, Xyn10D (both GH10), Xyn10Y, and Xyn10Z (both GH10 and CE1). The products released from wheat arabinoxylan after overnight reactions with these *C. thermocellum* xylanases were analysed by HPAEC-PAD for comparison with Xyn141E (Fig. 4). Hydrolysates produced by the GH10 xylanases generally show a larger diversity of peaks than do GH11 hydrolysates. Characteristic products of the GH10 family elute at retention times shortly after xylobiose (11.9 min and 13.2 min) and xylotriose (16.6 min and 17.2 min). In addition, GH10 enzymes produced a peak located between xylotetraose and xylopentaose (24.0 min), and arabinosylated oligosaccharides at retention times between 33 and 39 min. Xyn11A (GH11) did not produce these products. Instead, a prominent peak near the retention time of XA^3^XX was produced. GH10 hydrolysates also feature this peak, but the concentration tends to be lower. All GH10 and GH11 enzymes liberated xylose, xylobiose, xylotriose, and doubly-arabinosylated oligosaccharides (XA^2+3^XX and A^2+3^XX). The intensities of the individual peaks, and hence the product concentrations, varied among all enzymes, which can be explained by their different specificities. These observations coincide with previously described product patterns indicating that GH10 enzymes produce short arabinoxylo-oligosaccharides and that GH11 xylanases can only hydrolyse unsubstituted regions of the xylan backbone, leading to the accumulation of long-chain arabinoxylo-oligosaccharides^8, 14^. Compared to the typical GH10 and GH11 xylanases, Xyn141E showed a GH11-like product spectrum, as can be seen by the absence of the GH10 fingerprint peaks at retention times of 11.9 min, 13.2 min, 16.6 min, and 17.2 min. However, there are also some differences with the GH11 product spectrum. Most notably, the release of xylose cannot be detected (Fig. 4B). Additionally, Xyn141E shows a clear xylotetraose peak, which can only be seen in the product spectrum of Xyn10C, but not in that of Xyn11A. The xylotetraose peak of Xyn141E is preceded by some smaller peaks, corresponding to unknown substances that are also not present in the product pattern of Xyn11A. Thus, Xyn141E shows a GH11-like product spectrum but with some distinct differences.

### Substrate recognition based on hydrolysis of xylo- and arabinoxylo-oligosaccharides

To examine how Xyn141E recognises its substrates, the activity of Xyn141E on different commercially available xylo- and arabinoxylo-oligosaccharides (Supplementary Table S3) was tested in over-night reactions. The results are shown in Supplementary Figure S4. Xyn141E was able to hydrolyse X_5_ and X_4_ to X_2_ and X_3_ or X_2_, respectively. Only trace amounts of X_4_ (hydrolysis of X_5_) or X_3_ (hydrolysis of X_4_) and no xylose were detected. This indicates that XynE preferentially cleaves in the middle of oligosaccharide substrates, which agrees with the endo-mode of action observed on polysaccharides (Fig. 3). No activity on X_2_, X_3_ or any arabinosylated oligosaccharides was detected which means that Xyn141E recognises only unsubstituted regions of the xylan backbone and requires a minimum substrate chain length of four xylose residues.

## Discussion

The search for cellulases and hemicellulases in the genome of *C. thermocellum* has been the subject of intense research for more than 30 years. Initially, libraries of *C. thermocellum* gDNA were analysed by functional screenings for GHs in *E. coli* ^19, 32–35^. The first *C. thermocellum* xylanase was characterised in 1988, when Grépinet and colleagues cloned and examined Xyn10Z^20, 21^. In the following years, much work was invested in unravelling the enzymatic repertoire of *C. thermocellum*. Other GH10 and GH11 xylanases were characterised between 1995 and 2005^16, 17, 19, 36^. Studies on the cellulosome suggest a certain characteristic architecture shared by cellulosomal enzymes comprising catalytic GH-modules, dockerin modules and, in many cases, a CBM^24^. When the genomic sequence of *C. thermocellum* ATCC 27405 became available, it enabled the use of the dockerin module as a marker sequence for cellulosomal genes^25^. It became apparent that 12 of the 71 putative cellulosomal components of *C. thermocellum* contained components of unknown function in addition to DocI and CBM^24, 25^. We now are able to assign a function to one of these previously unknown components encoded by ORF cthe_2195. This ORF encodes an additional *C. thermocellum* endoxylanase, Xyn141E, which is unrelated to all other previously described xylanases.

With so much effort dedicated to the identification of active *C. thermocellum* enzymes in sequence-independent activity screenings, it is surprising that no activity has been reported for Xyn141E to date. One explanation may be the relatively low activity of Xyn141E against commercially available standard substrates in comparison to other xylanases. The specific xylanase activity of Xyn141E (92 mU/mg) is lower than those of the GH10 and GH11 xylanases of *C. thermocellum* by a factor of 180 (Xyn10Y: 16.5 U/mg) to 7,490 (Xyn11A: 689 U/mg)^16, 19^. It is therefore plausible to assume that Xyn141E was never detected in functional screenings of *C. thermocellum* gDNA libraries based on halo formation after Congo Red staining^20^. Although xylan was the preferred substrate of Xyn141E in this study, the main activity of Xyn141E may be directed against a substrate that was not yet tested. It is also possible that Xyn141E is converted into its naturally more active form by post-translational modification in *C. thermocellum*, which may not occur during heterologous expression in *E. coli*.

Interestingly, Xyn141E has not been detected by mass spectrometric analyses of *C. thermocellum* ATCC 27405 cellulosomes^25, 37^. ORF cthe_2195 is part of a presumed operon with three other genes (5’ cthe_2197-cthe_2196-cthe_2195-cthe_2194 3’) encoding enzymes with a known GH or CE-module, DocI, and CBM6^37^. The enzymes encoded by these three genes were also not detected. Raman *et al*. hypothesised that the operon might not have been induced under the experimental conditions chosen by Zverlov *et al*. and Raman *et al*.^25, 37^. Interestingly, Cthe_2197 was detected in a recent study of *C. thermocellum* DSM1313 mutants using a proteomics approach^38^. We detected a mobile element of the IS21 family that disrupts ORF cthe_2197 in *C. thermocellum* ATCC 27045 and does not occur in the corresponding ORF Clo1313_2861 in *C. thermocellum* DSM1313. A detailed characterisation of this mobile element is provided in Supplementary Table S5. *In silico* removal of the mobile element restores ORF cthe_2197 completely to the state of ORF Clo1313_2861, suggesting that the functional expression of the operon containing ORF cthe_2195 is obstructed in *C. thermocellum* ATCC 27405, explaining why Xyn141E was not detected in the functional screenings of *C. thermocellum* ATCC 27405 gDNA libraries. Considering the catalytic modules of Cthe_2194 (CE1, CBM6, DocI), Cthe_2196 (GH43, CBM6, DocI) and Cthe_2197 (GH2, CBM6, DocI), we could show involvement in hemicellulose degradation for two additional enzymes of the operon: in a qualitative assay, we found hydrolytic activities of purified Cthe_2196 against wheat arabinoxylan and oat spelt xylan and of purified Cthe_2197 against *para*-nitrophenyl-β-d-galactopyranoside.

Xyn141E is structurally distinct from enzymes in xylanase families GH10 and GH11; the right-handed beta-helix region predicted in the catalytic module differs considerably from the structures of the GH10 [(β/α)_8_-barrel] and GH11 (β-jelly roll) xylanases (CAZy database^6^). With a molecular mass of 102 kDa, Xyn141E is among the largest cellulosomal components identified as only 15 of the 71 cellulosomal components reported by Zverlov *et al*.^25^ are larger than or comparable in size to Xyn141E. Here, Xyn141E resembles GH10 xylanases, which are characterised by a high molecular mass in contrast to the low molecular mass of the GH11 xylanases^5^.

In addition to its structural differences to the GH10 and GH11 xylanases, Xyn141E is further distinguished by its catalytic properties. Based on its side activities (against CMC, BBG, and mannan), the substrate spectrum of Xyn141E is similar to those of GH10. In contrast, GH11 xylanases are solely active against xylose-containing substrates^5^.

While the product pattern from arabinoxylan suggests a similarity to that of GH11, particularly with regard to the absence of GH10-fingerprint peaks following xylobiose and xylotriose, further inspection reveals that Xyn141E is distinguished from GH11 by the inability to release xylose and the presence of xylotetraose in the product spectrum. Examination of the substrate recognition by Xyn141E using xylo- and arabinoxylo-oligosaccharides as substrates showed that Xyn141E did not hydrolyse arabinosylated oligosaccharides. Arabinose side chains probably prevent substrate recognition due to steric hindrance. The combination of a GH10-like substrate spectrum and a GH11-like product pattern suggests that the substrate-binding region and active centre of Xyn141E are structurally different from those of the GH10 and GH11 xylanases. It can therefore be hypothesised that Xyn141E has a catalytic mechanism that differs from those of both GH10 and GH11.

Shortly before submission of this manuscript, Ndeh *et al*.^7^ published the α-l-fucosidase BT1002 (Genbank accession number AAO76109.1) from *Bacteroides thetaiotaomicron*, which is the founding member of GH141. Although BT1002 is characterised as α-l-fucosidase^7^, Xyn141E did not show any activity on *p*NP-α-l-fucoside. It is interesting to observe that the first two members of the new GH141 family show distinctly different activities. A dendrogram comparing sequences deposited in GH141 reveals that Xyn141E and BT1002 (similarity: 33.4%, identity 20.7%, pairwise global alignment) are positioned at different branches and that Xyn141E might belong to a different subfamily of GH141 than BT1002 (Supplementary Figure S6). This can also explain the different activities of Xyn141E and BT1002, since enzymes from different GH subfamilies often also have different substrate specificities^39^.

A broad distribution and functional relevance of the new GH141 family are suggested by the abundance of bacterial species with proteins homologous to Xyn141E. However, to our knowledge, none of these homologous proteins have been characterised, and their biological function remains unknown. With xylanase Xyn141E, we have now characterised the first xylanase of the novel GH141 family. This knowledge will facilitate the analysis of additional GH141 enzymes to help define their biological significance, fill gaps in automated protein annotation, and assess potential industrial applications.

## Material and Methods

### Sequence analysis

#### Protein sequence alignments

Identification of homologous protein sequences and annotation of putative conserved modules were performed using blastp 2.5.0+^40, 41^.

### Construction of expression vector

*C. thermocellum* ATCC 27405 ORF cthe_2195 was synthesised and codon-optimised for expression in *E. coli*, and inserted into the pET24c(+) vector (Merck Millipore, Darmstadt, Germany) by Eurofins Genomics (Ebersberg, Germany) to yield the expression plasmid pET24c(+)-cthe2195syn. The synthesised gene encodes amino acids 40–965 of the Xyn141E protein. N-terminal amino acids 1–39 were excluded, because a putative signal peptide was predicted by SignalP4.1^26^. Additionally, the target protein was designed to carry a C-terminal His_6_-tag for purification by IMAC. The nucleotide sequence of the synthesised gene was submitted to the European Nucleotide Archive (accession number: LT718214, http://www.ebi.ac.uk/ena/data/view/LT718214).

### Production and purification of recombinant Xyn target proteins

#### Expression of *xynE*

Recombinant *xynE* was expressed using *E. coli* BL21 Star™ (Life technologies, Carlsbad, USA) transformed with the pET24c(+)-cthe2195syn expression plasmid. *E. coli* cells were grown in 2 × 250 mL ZYP-5052 autoinduction medium^42^ containing 50 μg/mL kanamycin. Each flask was inoculated with 10 mL of a 20 mL overnight starter culture and incubated at 37 °C with shaking at 180 rpm for 18 h. The reference *C. thermocellum* xylanases Xyn11A, Xyn10C, Xyn10D, Xyn10Y, and Xyn10Z were produced in the same manner.

#### Purification of recombinant protein

To isolate the target protein, the cells were harvested by centrifugation (3,440 × *g* for 10 min at 4 °C). The cell pellets were suspended in 20 mL of the supernatant and centrifuged once more (4,890 × *g* for 15 min at 4 °C). The cell pellets were resuspended in lysis buffer (50 mM MOPS, pH 7.3, 20 mM imidazole, 0.5 M NaCl, and 20 mM CaCl_2_), supplemented with Complete Mini EDTA-free protease inhibitor cocktail (Roche Diagnostics, Mannheim, Germany) and lysozyme (1 mg/mL), and incubated on ice for 30 min. Cell lysis was achieved by sonication for 2 × 4 min on ice with a pause of 2 min between the sonication steps to cool the sample. After removal of cell debris by centrifugation (38,500 × *g* for 20 min at 4 °C), the supernatant was applied to a 5 mL His-Trap FF column (GE Healthcare, Little Chalfont, GB), according to the supplier’s protocol. After washing with four column volumes of lysis buffer, the recombinant target protein was eluted with a mixture of 50% elution buffer (50 mM MOPS, pH 7.3, 0.5 M imidazole, 0.1 M NaCl, and 5 mM CaCl_2_) and 50% lysis buffer. For further purification of the target protein, residual *E. coli* proteins were denatured by heating of the eluate fraction at 60 °C for 20 min. The precipitate was removed by centrifugation (16,000 × *g* for 15 min at 4 °C). Final concentrations of 20% (w/v) glycerol and 0.02% (w/v) NaN_3_ were added to the purified enzyme, which was then stored at 4 °C.

#### Determination of concentration, size, and purity

The concentrations of the purified proteins were determined by measuring extinction at 280 nm under denaturing conditions (8 M urea), using the extinction coefficient calculated by the ExPASy ProtParam Tool (http://web.expasy.org/tools/protparam/, assuming all cysteine residues are reduced). Verification of the apparent molecular mass and purity of the protein was assessed by SDS-PAGE by the method of Laemmli (1970) using 10% gels.

### Enzyme assays using polysaccharide substrates

#### Analysis of substrate specificity

To examine the substrate specificity of Xyn141E, each polysaccharide substrate was incubated with 4 µg/mL Xyn141E in standard reaction buffer (100 mM MOPS, pH 6.5, 50 mM NaCl, and 10 mM CaCl_2_) at 60 °C for 20 h in a total reaction volume of 150 µL in 96-well PCR plates. The pH of all buffers were adjusted at the temperature of the respective experiments (e.g., 60 °C). The substrates and their final concentrations are listed in Supplementary Table S1. For the quantification of the specific activity of Xyn141E against wheat arabinoxylan, birch wood xylan, oat spelt xylan, 4-*O*-methyl-glucuronoxylan, CMC, BBG, and ivory nut mannan, the Xyn141E concentration was increased to 66.7 µg/mL and the incubation time was reduced to 60 min. A 50 µL aliquot of the reaction mix was used for quantification of reducing sugars released from the polymeric substrates, according to the method of Wood and Bhat^43^, using 3,5-dinitrosalicylic acid (DNSA). A sigmoidal calibration curve was determined using known concentrations of standard glucose solutions. One enzymatic unit [U] was defined as the amount of enzyme that liberates one µmol glucose equivalent per minute. All assays were performed in triplicate. The activity of Xyn141E on 13 different *para*-nitrophenol-glycosides (Supplementary Table S2) was tested by incubation of 4 µg/mL Xyn141E in standard reaction buffer at 60 °C for 15 h and 21 h (total reaction volume: 500 µL). The assay was performed as described in^12^. The hydrolysis of commercially available xylo- and arabinoxylo-oligosaccharides was tested by incubating 200 µg/mL of substrate (Supplementary Table S3) with 100 µg/mL Xyn141E in 10 mM MOPS, pH 6.5, 50 mM NaCl, 10 mM CaCl_2_ at 60 °C for 16 h in a total reaction volume of 100 µL. The analysis of the hydrolysis products was performed by HPAEC-PAD (see below).

#### Examination of physicochemical properties

Ten micrograms of Xyn141E were used in a total volume of 150 µL containing 0.5% (w/v) soluble wheat arabinoxylan. For determination of the activity-pH profile, 100 mM sodium acetate buffer (at pH 5.0, 5.5, and 6.0) or 100 mM MOPS buffer (at pH 6.0, 6.5, 7.0, and 7.5) supplemented with 50 mM NaCl and 10 mM CaCl_2_ was used. The enzyme reaction was carried out for 2 h at 65 °C. For the determination of the temperature dependence of Xyn141E, 67 mM MOPS (pH 6.5) containing 33 mM NaCl and 6.7 mM CaCl_2_ was used and the reaction was incubated at temperatures ranging from 50 °C–75 °C and from 64 °C–90 °C for 2 h in 96-well PCR plates, using a gradient PCR cycler (T-Advanced 96 G, Biometra GmbH, Göttingen, Germany). The influence of salts (CaCl_2_, MgCl_2_, and CuSO_4_), EDTA, SDS, and ethanol on Xyn141E was analysed using 100 mM MOPS buffer pH 6.5, supplemented with these compounds at concentrations ranging from 1 mM – 10 mM (salts, EDTA, and SDS) or 1–5% (w/v; ethanol) and an incubation time of 60 min at 65 °C. The released reducing sugars were quantified using the DNSA method^43^, as described above. All assays were performed in triplicate. Product formation over time was analysed by incubating 200 µg/mL Xyn141E with 0.5% (w/v) wheat arabinoxylan in standard reaction buffer at 65 °C. At selected time points, aliquots were taken and the reaction was stopped by boiling the samples for 10 min in a 95 °C water bath. The products were analysed by HPAEC-PAD.

### Analysis of polysaccharide degradation products

For high-performance anion-exchange chromatography with pulsed amperometric detection (HPAEC-PAD), the ICS-3000 chromatography system (Dionex Softtron GmbH, Germering, Germany), a CarboPac PA1 4 × 50 mm pre-column, and a CarboPac PA1 4 × 250 mm main column were used. The eluent gradient profile used for analyte separation began at 7.5 mM sodium acetate (NaOAc) with 100 mM NaOH at 0 min and linearly increased to 100 mM NaOAc with 100 mM NaOH at 67.5 min. To wash the column, the concentration of NaOAc was increased to 650 mM NaOAc with 100 mM NaOH for 4 min and subsequently re-equilibrated with 100 mM NaOH for 16.3 min after each run. Carbohydrate detection by PAD was based on the waveform “standard carbohydrate quad,” which was set to 1 Hz. Prior to the analysis of polysaccharide hydrolysates by HPAEC-PAD, the samples were diluted 10 × with ddH_2_O and supplemented with 2 mg/L d- mannitol (final concentration) as an internal standard to monitor the stability of the detector signal. Commercially available xylo- and arabinoxylo-oligosaccharides (Megazyme, Bray, Ireland) were used as reference substances for the identification of the products: xylobiose, -triose, -tetraose, -pentaose (X_2_–X_5_), 3^2^-α-l-arabinofuranosyl-xylobiose (A^3^X), 2^3^-α-l-arabinofuranosyl-xylotriose (A^2^XX), 2^3^-α-l-arabinofuranosyl-xylotetraose (XA^2^XX), 3^3^-α-l-arabinofuranosyl-xylotetraose (XA^3^XX), 2^3^,3^3^-di-α-l-arabinofuranosyl-xylotetraose (XA^2+3^XX), and 2^3^,3^3^-di-α-L-arabinofuranosyl-xylotriose (A^2+3^XX). The nomenclature used for the arabinoxylo-oligosaccharide standards is in accordance with published standards^31^.

## Electronic supplementary material

Supplementary Information
