# Supplementary material for: Identification of endoxylanase XynE from Clostridium thermocellum as the first xylanase of glycoside hydrolase family GH141
Source: Sci Rep. 2017 Sep 11;7:11178. doi: 10.1038/s41598-017-11598-y (PMC5593877; doi:10.1038/s41598-017-11598-y)
Supplement: Supplementary file 1 — Supplementary Information [file 41598_2017_11598_MOESM1_ESM.pdf]

Supplementary Information

Identification of endoxylanase XynE from *Clostridium thermocellum* as the first xylanase of glycoside hydrolase family GH141

Simon Heinze[a], Matthias Mechelke[a], Petra Kornberger[a], Wolfgang Liebl[a,*], Wolfgang H. Schwarz[a], Vladimir V. Zverlov[a,b,*]

[a] Department of Microbiology, Technical University of Munich, Emil-Ramann-Str. 4, D-85354 Freising-Weihenstephan, Germany

[b] Institute of Molecular Genetics, Russian Academy of Science, Kurchatov Sq. 2, 123182 Moscow, Russia

* To whom correspondence should be addressed: vladimir.zverlov@tum.de, wliebl@wzw.tum.de

Supplementary table S1: Natural polysaccharide substrates tested and their final concentration

Substrate	Concentration [%, (w/v)]	Source
Arabinan (sugar beet)	0.5	Megazyme (Bray, Ireland)
Arabinogalactan (larch)	0.5	Megazyme
Arabinoxylan (wheat, medium viscosity)	0.5	Megazyme
Barley-β-glucan (medium viscosity)	0.5	Megazyme
Curdlan	0.5	Serva (Heidelberg, Germany)
Galactan (lupin)	0.5	Megazyme
Galactan (potato)	0.5	Megazyme
Gummi arabicum (acacia)	0.5	Sigma-Aldrich (St. Louis, USA)
Inulin (Dahlia tubers)	0.5	Sigma-Aldrich
Laminarin	0.5	Alfa Aesar (Karlsruhe, Germany)
Levan (*Serratia levanicum*)	0.25	Sigma-Aldrich
Lichenan	0.5	Sigma-Aldrich
Mannan (ivory nut)	0.75	Megazyme
Pachyman	0.5	Megazyme
Pectic galactan (lupin)	0.5	Megazyme
Pectic galactan (potato)	0.5	Megazyme
Polygalacturonic acid	0.5	Sigma-Aldrich
Pullulan	0.5	ICN Biochemicals (Irvine, USA)
Rhamnogalacturonan I	1.0	Megazyme
Sinistrin	0.5	Fresenius Kabi Austria GmbH (Graz, Austria)
4-O-Methyl-glucuronoxylan	0.5	Sigma-Aldrich
Xylan (birch, hardwood)	1.0	Sigma-Aldrich
Xylan (oat spelt)	0.5	Sigma-Aldrich
Xylan (larch, Softwood)	0.5	ICN Biochemicals
Xyloglucan	0.5	Megazyme
Chitosan	0.5	Sigma-Aldrich
Avicel	0.5	Serva
PASC	0.5	Prepared from Avicel [1]
CMC	0.5	Sigma-Aldrich

Supplementary table S2: *para*-Nitrophenyl (*p*NP-) substrates tested. The final concentration was 2 mM in all cases.

**Substrate**	**Source**
*p*NP-α-L-arabinofuranoside	Carbosynth Limited (Compton, Berkshire, UK)
*p*NP-α-L-fucopyranoside	Carbosynth
*p*NP-β-L-fucopyranoside	Carbosynth
*p*NP-α-D-glucopyranoside	Sigma-Aldrich
*p*NP-β-D-glucopyranoside	Carbosynth
*p*NP-α-D-galactopyranoside	Serva
*p*NP-β-D-galactopyranoside	Carbosynth
*p*NP-α-D-mannopyranoside	Carbosynth
*p*NP-β-D-mannopyranoside	Carbosynth
*p*NP-α-L-rhamnopyranoside	Carbosynth
*p*NP-α-D-xylopyranoside	Carbosynth
*p*NP-β-D-xylopyranoside	Sigma-Aldrich
*p*NP-β-D-cellobioside	Sigma-Aldrich

Supplementary table S3: XOS and AXOS mentioned in this study. Nomenclature with minor modifications adapted from [2,3]. Blue hexagon: xylose, orange pentagon: arabinose. The rightmost hexagon symbol represents the reducing-end xylose residue. The oligosaccharides listed here (purchased from Megazyme) were also used as substrates in experiments to examine the substrate specificity of Xyn141E (final concentration 200 µg/mL), except X and A^{2+3}XX. Table adopted with changes from [4].

**Name**	**Schematic structure**
**X,X$_2$-X$_5$** (xylose, xylobiose, -triose, tetraose, pentaose)	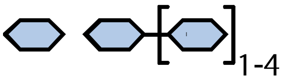
**A^{3}X** (3^2-a-L-arabinofuranosyl-xylobiose)	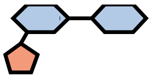
**A^{2}XX** (2^3-a-L-arabinofuranosyl-xylotriose)	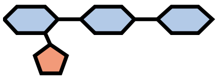
**XA^{2}XX** (2^3-a-L-arabinofuranosyl-xylotetraose)	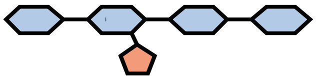
**XA^{3}XX** (3^3-a-L-arabinofuranosyl-xylotetraose)	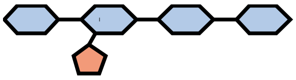
**XA^{2+3}XX** (2^3,3^3-di-a-L-arabinofuranosyl-xylotetraose)	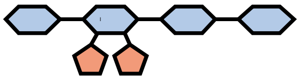
**A^{2+3}XX** (2^3,3^3-di-a-L-arabinofuranosyl-xylotriose)	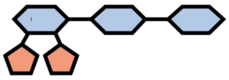

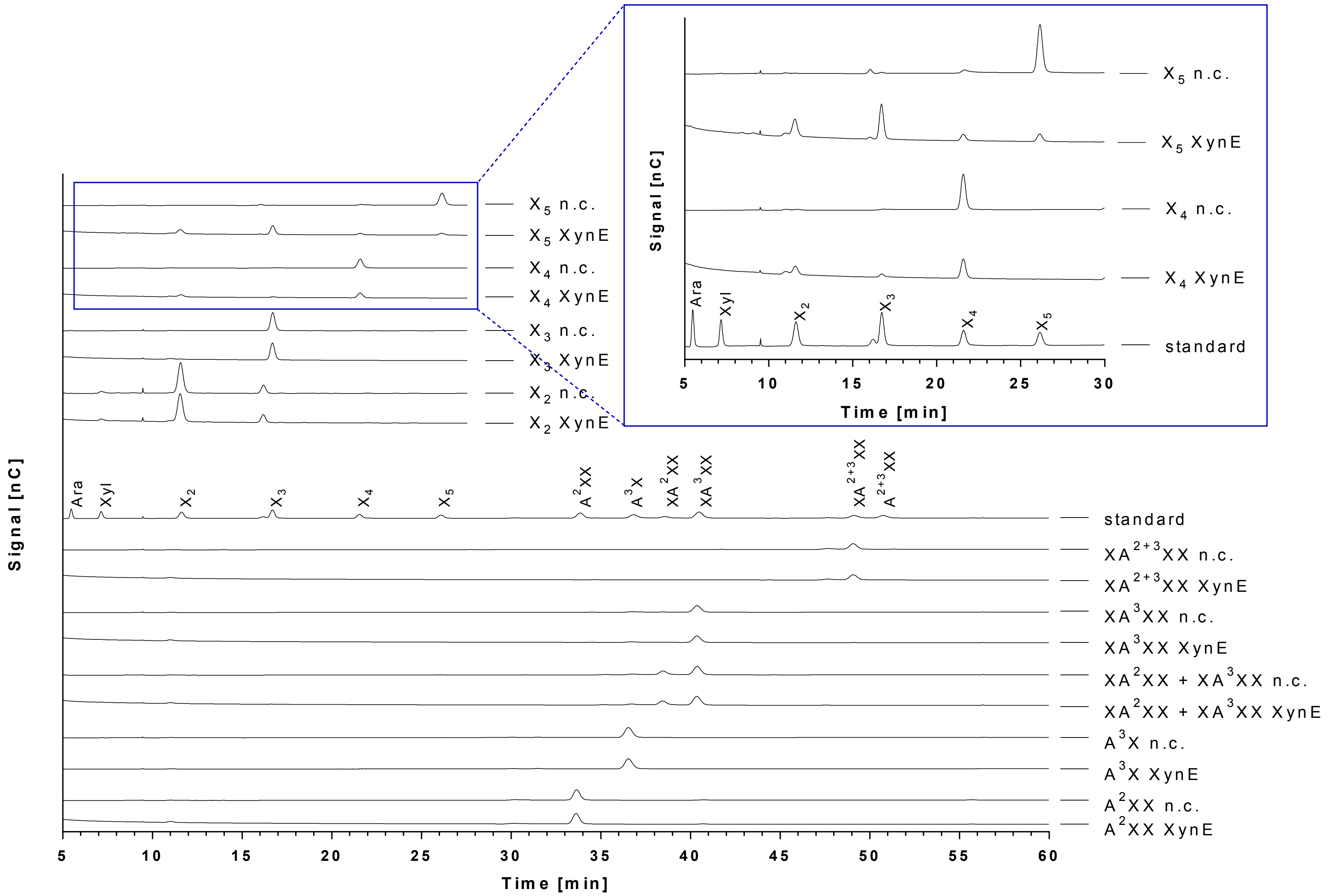

Supplementary figure S4: Hydrolysis of xylo-oligosaccharides and arabinoxylo-oligosaccharides by Xyn141E. 200 µg/mL of commercially available xylo-oligosaccharides and arabinoxylo-oligosaccharides were incubated with 100 µg/mL Xyn141E in 10 mM MOPS, pH 6.5, 50 mM NaCl, 10 mM $CaCl_2$ at 60 °C for 16 h in a total reaction volume of 100 µL. The analysis of the hydrolysis products was performed by HPAEC-PAD. For nomenclature of the oligosaccharides see supplementary table S3. n.c. = negative control, no addition of Xyn141E. Inset: zoom into the relevant parts of the chromatograms for the degradation of X_4 and X_5.

Supplementary table S5: Characteristics of the mobile element (IS21 family) disrupting ORF cthe_2197 in *C. thermocellum* ATCC 27405 compared to typical features of the IS21 family, according to Mahillon and Chandler [5].

	C. thermocellum **ATCC 27405**	**Typical Features for IS21 Family Mobile Elements**
Genomic location of IS21 disrupting ORF cthe_2197	2625557–2627975	-
Genomic location of additional copies of IS21	2383571–2385989<br>2726668–2729086<br>3345980–3348398	-
Length	2419 bp	1950–2500 bp
Direct target repeat in ORF cthe_2197	AGGTTTTT	-
Length of direct target repeat	8 bp	Usually 4 bp, sometimes 5 bp or 8 bp
Terminal base pairs	TG	TG
Terminal inverted repeats	TGTTAATG	yes
Number of ORFs	2	2
Other species with a similar mobile element	*Clostridium clariflavum* DSM 19732<br>2 matches, 85% identity	-

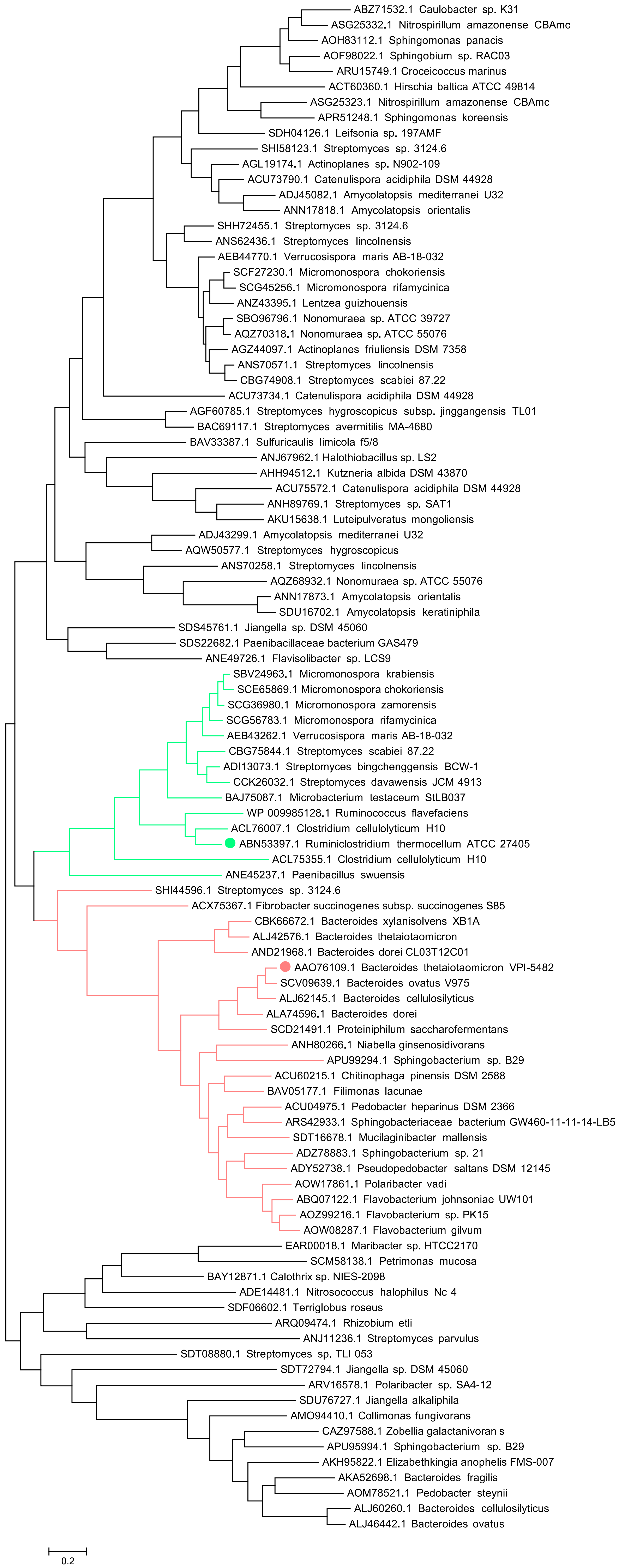

Supplementary figure S6: The best-scoring maximum-likelihood (ML) tree of family GH141. The sequences of GH141 family proteins were obtained by the NCBI GenBank IDs deposited in the CAZy database. For tree construction, the 147 sequences in family GH141 were reduced to 99 by clustering them via the UCLUST algorithm[6] and the resulting sequences were aligned using Clustal Omega[7]. Phylogenetic analysis was conducted using the Maximum Likelihood method based on the Poisson correction model[8] in MEGA7[9]. Initial trees for the heuristic search were generated by applying Neighbor-Join and BioNJ algorithms to a matrix of pairwise distances estimated using a JTT model, and then selecting the topology with superior log likelihood value. The tree with the highest inferred log likelihood is shown. The tree is drawn to scale, with branch lengths measured in the number of substitutions per site. All leaves are labelled by their GenBank accession numbers and species names. The leaves for BT1002 from *Bacteroides thetaiotaomicron* VPI-5482 and Xyn141E from *Clostridium thermocellum* ATCC 27405 are labelled with pink and green circles, respectively.

Supplementary References

1. Wood, T. M. Preparation of crystalline, amorphous, and dyed cellulase substrates. *Methods Enzymol.* **160,** 19–25 (1988).

2. McCleary, B. V. *et al.* Hydrolysis of wheat flour arabinoxylan, acid-debranched wheat flour arabinoxylan and arabino-xylo-oligosaccharides by β-xylanase, α-l-arabinofuranosidase and β-xylosidase. *Carbohydr. Res.* **407,** 79–96 (2015).

3. Fauré, R. *et al.* A Brief and Informationally Rich Naming System for Oligosaccharide Motifs of Heteroxylans Found in Plant Cell Walls. *Aust. J. Chem.* **62,** 533 (2009).

4. Mechelke, M. *et al.* Characterization of the arabinoxylan-degrading machinery of the thermophilic bacterium Herbinix hemicellulosilytica—Six new xylanases, three arabinofuranosidases and one xylosidase. *J. Biotechnol.* (2017). doi:10.1016/j.jbiotec.2017.04.023

5. Mahillon, J. & Chandler, M. Insertion sequences. *Microbiol. Mol. Biol. Rev.* **62,** 725–774 (1998).

6. Edgar, R. C. Search and clustering orders of magnitude faster than BLAST. *Bioinformatics* **26,** 2460–2461 (2010).

7. Li, W. *et al.* The EMBL-EBI bioinformatics web and programmatic tools framework. *Nucleic Acids Res.* **43,** W580-4 (2015).

8. Zuckerkandl, E. & Pauling, L. in *Evolving Genes and Proteins* 97–166 (1965). doi:10.1016/B978-1-4832-2734-4.50017-6

9. Kumar, S., Stecher, G. & Tamura, K. MEGA7: Molecular Evolutionary Genetics Analysis Version 7.0 for Bigger Datasets. *Mol. Biol. Evol.* **33,** 1870–1874 (2016).
